# Externalizing Behaviors and Callous-Unemotional Traits: Different Associations With Sleep Quality

**DOI:** 10.1093/sleep/zsx070

**Published:** 2017-06-01

**Authors:** Dan Denis, Reece Akhtar, Benjamin C Holding, Christina Murray, Jennifer Panatti, Gordon Claridge, Avi Sadeh, Nicola L Barclay, Rachael O’Leary, Barbara Maughan, Tom A McAdams, Richard Rowe, Thalia C Eley, Essi Viding, Alice M Gregory

**Affiliations:** 1 Center for Sleep and Cognition, Beth Israel Deaconess Medical Center, Harvard Medical School, Boston, MA;; 2 King's College London, MRC Social, Genetic, and Developmental Psychiatry Centre, Institute of Psychiatry, Psychology, and Neuroscience, London, UK;; 3 Department of Psychology, University of Sheffield, Sheffield, UK;; 4 Division of Psychology and Language Sciences, University College London, London, UK;; 5 Department of Clinical Neuroscience, Karolinska Institutet, Stockholm, Sweden;; 6 Department of Psychology, Goldsmiths, University of London, London, UK;; 7 Department of Psychology, Oxford University, Oxford, UK;; 8 School of Psychological Sciences, Tel Aviv University, Tel Aviv, Israel;; 9 Sleep and Circadian Neuroscience Institute, Nuffield Department of Clinical Neurosciences, University of Oxford, Oxford, UK

**Keywords:** actigraphy, antisocial, callous-unemotional, externalizing, psychopathology, sleep

## Abstract

**Study Objectives:**

Sleep quality is associated with different aspects of psychopathology, but relatively little research has examined links between sleep quality and externalizing behaviors or callous-unemotional traits. We examined: (1) whether an association exists between sleep quality and externalizing behaviors; (2) whether anxiety mediates this association; (3) whether callous-unemotional traits are associated with sleep quality.

**Methods:**

Data from two studies were used. Study 1 involved 1556 participants of the G1219 study aged 18–27 years (62% female). Questionnaire measures assessed sleep quality, anxiety, externalizing behaviors, and callous-unemotional traits. Study 2 involved 338 participants aged 18–66 years (65% female). Questionnaires measured sleep quality, externalizing behaviors, and callous-unemotional traits. In order to assess objective sleep quality, actigraphic data were also recorded for a week from a subsample of study 2 participants (*n* = 43).

**Results:**

In study 1, poorer sleep quality was associated with greater externalizing behaviors. This association was partially mediated by anxiety and moderated by levels of callous-unemotional traits. There was no significant relationship between sleep quality and callous-unemotional traits. In study 2, poorer sleep quality, as assessed via self-reported but not objective measures, was associated with higher levels of externalizing behaviors. Furthermore, in study 2, better sleep quality (indicated in both questionnaires and actigraphy measures: lower mean activity, and greater sleep efficiency) was associated with higher levels of callous-unemotional traits.

**Conclusions:**

Self-reports of poorer sleep quality are associated with externalizing behaviors, and this association is partially mediated by anxiety. Callous-unemotional traits are not associated with poor sleep and may even be related to better sleep quality. This is an exceptional finding given that poor sleep quality appears to be a characteristic of most psychopathology.

**Statement of Significance**

Sleep quality has been linked to different types of psychopathology, but associations with externalizing behaviors are relatively underexplored. In this study, we report moderate associations between reports of poorer sleep quality and greater externalizing behaviors in two separate samples. More novel is that the association appears to be partially mediated by anxiety. Most uniquely, we show, for the first time, and in two separate samples, that a subtyping variable for conduct disorder, callous-unemotional traits, do not appear to be associated with poorer sleep. In fact, self-reported and objective data from study 2 indicates an association between higher levels of callous-unemotional traits and better sleep quality. This latter finding provides a timely reminder that further research is necessary on the specificity of associations between sleep and different forms of psychopathology.

## INTRODUCTION

A small but growing body of work has demonstrated associations between externalizing behaviors, such as aggressive and rule-breaking behaviors, and poor sleep quality.^1–3^ There are multiple possible explanations for such associations. For example, poor sleep quality may lead to reduced ability to regulate emotions^4^ which could potentially lead to emotionally charged aggression. Conversely, externalizing behaviors could disturb sleep via mechanisms such as ruminating and catastrophizing about these behaviors—which could disrupt sleep.^5^ Sleep problems have been shown to be comorbid with phenotypes such as impulse-control disorders, that is, attention-deficit hyperactivity disorder and substance use disorders,^3^ which may play a mediating role in the link between externalizing behaviors and sleep quality.^6^

Another variable that may contribute to the association between externalizing behaviors and poor sleep quality is anxiety. Anxiety and poor sleep quality commonly co-occur.^7^ Furthermore, it has been shown that those with externalizing behaviors frequently show co-occurring anxiety symptoms or full anxiety disorders,^8,9^ and many children with conduct problems are hypervigilant to possible threat.^10^ Such anxious presentation may mediate, in part, the association between externalizing behaviors and poor sleep quality in some individuals, but this possibility has not been thoroughly examined in the past.

A particular group of individuals who often show persistent and severe externalizing behaviors are those who exhibit callous-unemotional traits (termed as “Limited Prosocial Emotions” within the fifth edition of the Diagnostic and Statistical Manual of Mental Disorders [DSM-5]).^11^ Callous-unemotional traits include a lack of empathy, indifference toward the feelings of others, and lack of emotional reactivity—and represent a diagnostic specifier for conduct disorder in DSM-5^11^. As well as increased risk for persistent and severe antisocial conduct, callous-unemotional individuals show vulnerability to other psychopathological outcomes.^12^ Despite these traits being an indicator for maladaptive long-term outcomes, it has recently been hypothesized that callous-unemotional traits may not be associated with sleep problems.^13^ It is possible that low levels of emotional reactivity and anxiety experienced by those with callous-unemotional traits may be protective against sleep complaints. Indeed, high levels of callous-unemotional traits are typically associated with low anxiety, attenuated reactivity of the brain’s affective circuits to emotional stimuli and the tendency to externalize blame.^12^

Another possibility is that levels of callous-unemotional traits may moderate the relationship between externalizing behaviors and sleep quality. For the aforementioned reasons, it is possible that the association between externalizing behaviors and sleep quality is weaker in those with high as compared to low levels of callous-unemotional traits. However, the association between callous-unemotional traits and sleep problems has not been examined to date. Despite callous-unemotional traits being typically thought of as being inversely related to anxiety,^12^ some data indicate that it is possible for callous-unemotional traits and anxiety symptoms to co-occur.^14^ For example, a recent study showed no differences in callous-unemotional traits between individuals with conduct disorder with a comorbid anxiety disorder compared to those with a conduct disorder without a comorbid anxiety disorder.^9^ It has been suggested that two forms of callous-unemotional traits may exist—those with and those without anxiety or comorbid internalizing symptoms, and the two forms are thought to have different origins with the former influenced to a greater extent by a history of childhood adversity, and the latter influenced to a greater extent by genetic vulnerabilities.^12,14^

The current study examines the association between externalizing behaviors and sleep problems in two different samples and with both the self-report Pittsburgh Sleep Quality Index^15^ and an objective (actigraphy^16^) measure of sleep quality (in a subsample [*n* = 43] of participants in study 2 only). Furthermore, we assessed whether anxiety mediates the association between externalizing behaviors and sleep. We also tested whether the relationship between externalizing behaviors and sleep quality was moderated by levels of callous-unemotional traits. Based on the extant literature we hypothesized that: (1) externalizing behaviors will be associated with poorer sleep quality; (2) the association between externalizing behaviors and poorer sleep quality will be partly mediated by anxiety; (3) callous-unemotional traits will not be associated with sleep quality; and (4) the relationship between externalizing behaviors and poor sleep quality will be moderated by levels of callous-unemotional traits. Two independent studies addressing these questions are presented below.

## STUDY 1

### Methods

#### Participants

Participants were from wave 4 of the G1219 longitudinal twin/sibling study. G1219 initially comprised adolescent offspring of adults enrolled in a large-scale population-based study (GENESiS).^17^ The G1219 twins are a random selection of live twin births born between 1985 and 1988 identified by the UK Office of National Statistics. At wave 1 of data collection (which took place between 1999 and 2002), 3640 respondents aged between 12 and 19 years participated in the study. Informed consent was obtained from parents/guardians of all adolescents under 16 years and from the adolescents themselves when 16 years and over. Ethical approval for different stages of this study has been provided by the Research Ethics Committees of the Institute of Psychiatry, South London and Maudsley NHS Trust, and Goldsmiths, University of London. At wave 4 (which took place in 2007 and is the focus of this current report), we traced participants who had taken part in wave 2/3 and sent them a questionnaire booklet. A total of 1556 individuals (957 female) were included in the wave 4 data set (61% of those targeted; 74% of those participating at wave 3) presented in this study, aged between 18 and 27 years (mean [M] = 20.3, standard deviation [SD] = 1.76). Wave 4 is the only assessment at which callous-unemotional traits have been assessed and is hence the focus of this report.

## MEASURES

### Adult Self-Report: Externalizing Behaviors

Externalizing behaviors were measured by self-report using the aggression and rule-breaking subscales of the “Adult Self Report” scale.^18^ The item “I don’t feel guilty after doing something I shouldn’t” was removed from our analyses as the item content overlaps with the construct of callous-unemotional traits. Also, the item “I have trouble keeping a job” was not included because it was deemed it may be irrelevant to some of the participants given their age. Higher scores indicate increased externalizing behaviors. The final scale had 24 items and good internal reliability (*α* = 0.85).

### Inventory of Callous-Unemotional Traits

Eleven items from the inventory of callous-unemotional traits were used to measure callous-unemotional traits.^19^ In order to avoid overburdening the participants, a shortened version of the original scale (originally 24 items) was used, including the highest loading items from the callousness subscale (six items) and the unemotional subscale (five items). A global score was calculated using these items, where higher scores indicate increased callous-unemotional traits. The scale showed modest internal reliability (*α* = 0.64).

### Pittsburgh Sleep Quality Index

This 18-item self-report instrument measures sleep quality over the previous month across seven components of sleep (self-reported sleep quality, habitual sleep efficiency, sleep duration, sleep latency, sleep disturbance, daytime dysfunction, and use of sleep medications).^15^ The scale is validated for use in nonclinical samples.^20^ A global score of sleep quality is calculated by summing together responses from the seven components. Higher scores indicate poorer self-reported sleep quality. The internal reliability was good (*α* = 0.71).

### The Revised Symptoms of Anxiety Scale—Adapted

A 36-item questionnaire was used to yield a total anxiety score (Willis, unpublished), see the study by Gregory et al.^21^ for usage of this measure in other research. This is an age-appropriate version of the Revised Child Anxiety and Depression Scale.^22^ Participants were asked how often each item happens to them. A higher score indicates greater levels of anxiety. The scale had excellent internal reliability (*α* = 0.94).

### Statistical Analysis

Preliminary data screening found the externalizing behaviors (skew = 1.61) and anxiety symptoms (skew = 1.18) measures to be slightly skewed. Log transformations moderately improved the skew of both the externalizing behaviors (skew after log transform = −0.27) and anxiety symptoms (skew after log transform = −0.87) measures. As no major differences occurred when analyses were run on transformed variables, results on the raw (untransformed) data are reported here to maximize comparison with work by others. Analyses were conducted using Stata 9. When conducting analyses using G1219 data, the clustering option was used^23^ to account for the nonindependence of data obtained from twins. Multiple regression was performed to test the hypotheses that externalizing behaviors would be associated with poor sleep quality but that callous-unemotional traits would not. A bootstrap-mediated regression model was performed to test whether anxiety levels mediated the relationship between externalizing behaviors and sleep quality. Bootstrapping was used due to the increase in statistical power and does not assume normally distributed data.^24^ This was run using the PROCESS macro for SPSS,^24^ and nonindependence of errors was controlled for by randomly selecting one twin from each pair. Finally, an externalizing behaviors X callous-unemotional traits interaction term was added to the model to test whether the relationship between externalizing behaviors and sleep quality was moderated by the degree of callous-unemotional traits. Age and sex were controlled in all analyses by including them as predictors in regression models.

## RESULTS

### Descriptive Statistics and Correlations

Descriptive statistics and correlations between variables are shown in Table 1. There was a significant positive association between externalizing behaviors and callous-unemotional traits (*r* = 0.22, *p* < .001). Poorer sleep quality was significantly positively associated with externalizing behaviors (*r* = 0.32, *p* < .001) but not with callous-unemotional traits (*r* = 0.04, *p* = .17). Anxiety symptoms were significantly associated with poorer sleep quality (*r* = 0.40, *p* < .001) and externalizing behaviors (*r* = 0.27, *p* < .001) but not callous-unemotional traits (*r* = −0.03, *p* = .16).

**Table 1:** Descriptive Statistics and Correlations for Key Variables in Study 1.

	Variable	Mean	SD	Correlations		
				1	2	3
1	Externalizing behaviors	6.20	5.38			
2	Callous-unemotional traits	6.01	2.93	0.22***		
3	Self-reported sleep quality	5.70	3.01	0.32***	0.04	
4	Anxiety symptoms	25.07	14.88	0.27***	−0.04	0.40***

SD = standard deviation.

Externalizing behaviors (adult self-report) possible range = 0–50. Higher score = higher externalizing behaviors.

Callous-unemotional traits (inventory of callous-unemotional traits) possible range = 0–33. Higher score = higher callous-unemotional traits.

Self-reported sleep quality (Pittsburgh Sleep Quality Index) possible range = 0–21. Higher score = poorer sleep.

Anxiety symptoms (revised symptoms of anxiety scale) possible range = 0–108. Higher score = more anxiety symptoms.

***p < .001.

### Regression Analyses

A multiple regression model predicting self-reported sleep quality from externalizing behaviors and callous-unemotional traits accounted for a significant proportion of variance in sleep quality, after controlling for age and sex [overall model: *F*(4, 879) = 34.80, *p* < .001, *R*^2^ = 0.11]. Higher scores on the externalizing behaviors measure were associated with poorer self-reported sleep quality; *β* = 0.33, 95% confidence intervals (CI) = 0.28 to 0.39, *p* < .001. Callous-unemotional traits were not associated with self-reported sleep quality; *β* = −0.02, 95% CI = −0.08 to 0.03, *p* = .41.

A bootstrap-mediated regression model [number of samples = 5000; *F*(4, 710) = 49.74, *p* < .001, *R*^2^ = 0.22] showed that anxiety mediated the relationship between externalizing behaviors and poor sleep quality (indirect effect, *β* = 0.11, 95% CI = 0.07 to 0.15). Mediation was only partial, as externalizing behaviors remained statistically significant once anxiety was included in the model (Table 2).

**Table 2:** Bootstrapped Mediated Regression: Anxiety As a Mediator of the Association Between Externalizing Behaviors (Independent Variable) and Self-Reported Sleep Quality (Dependent Variable).

Variable	*β*	95% CI
Model 1: DV = anxiety symptoms *F*(3, 711) = 41.02***		
Externalizing behaviors	0.29***	0.22 to 0.35
Age	0.02	−0.02 to 0.06
Sex	0.56***	0.43 to 0.70
Model 2: DV = self-reported sleep quality *F*(4, 710) = 49.74***		
Anxiety symptoms	0.37***	0.29 to 0.44
Externalizing behaviors^a^	0.22***	0.15 to 0.28
Age	0.01	−0.03 to 0.05
Sex	−0.11	−0.25 to 0.02
Indirect effect		Bootstrapped 95% CI
Anxiety symptoms^b^	0.11	0.07 to 0.15

CI = confidence intervals; DV = dependent variable.

^a^Direct effect of externalizing behaviors on self-reported sleep quality.

^b^Indirect effect of externalizing behaviors on self-reported sleep quality, with anxiety symptoms as the mediator. Sex coded as 1 = male, 2 = female.

***p < .001.

An interaction between externalizing behaviors and callous-unemotional traits predicted self-reported sleep quality; *β* = −0.05, 95% CI = −0.09 to −0.01, *p* < .05. A simple slope analysis showed that when callous-unemotional traits were low (1 SD below the mean), the relationship between externalizing behaviors and poor self-reported sleep quality was stronger; *β* = 0.40, and the slope was significantly different from zero; *t* = 10.76, *p* < .001. When callous-unemotional traits were high (1 SD above the mean), the relationship between externalizing behaviors and poor self-reported sleep quality was weaker; *β* = 0.31, and the slope was still significantly different from zero; *t* = 11.16, *p* < .001. The fact that all the slopes were significantly different from zero suggests that at all levels of the moderator, externalizing behaviors still significantly predicted poorer self-reported sleep quality.

## STUDY 2

### Participants

The sample consisted of 338 (221 female) participants, aged 18–66 years (*M* = 23.5, SD = 8.2; 84% aged between 18–28 years), who completed an online questionnaire. A smaller subset of these participants (*n* = 43, 10 males) also had their sleep assessed using actigraphy. Actigraphy participants were all university students aged between 18 and 30 years (*M* = 20.9, SD = 3.1).

### Measures

#### Adult Self-Report: Externalizing Behaviors

The same externalizing behavior items from the adult self-report reported in study 1 were included in these analyses. Here, the scale showed good internal reliability (*α* = 0.85).

#### Levenson’s Self-Report of Psychopathy Scale

The Levenson’s scale was used here and measures psychopathy in the nonclinical population.^25^ The self-report scale consists of 26 items that produces two subscales. Here we used all 16 items from the subscale measuring callous-unemotional traits (*α* = 0.63).

#### Pittsburgh Sleep Quality Index

The Pittsburgh Sleep Quality Index was used to assess sleep quality. See Study 1 for details. The scale showed good internal reliability (*α* = 0.71).

#### Actigraphy

Actigraphy provides an objective measure of sleep quality.^26–28^ By wearing a small device (a ‘MicroMini Motionlogger’, made by Ambulatory Monitoring, Inc.) on the wrist of their nondominant hand for 7 days, participant’s physical activity during sleep was measured. Data were averaged to obtain mean scores for the whole week. The actigraphy data were scored and analyzed using the “Cole-Kripke” algorithm in a program called “Action-W”. Raw data were recorded in 60-second epochs, with the movement sampling mode utilized with zero-crossing mode. Four trained raters used sleep diary data to detect and remove artifacts from the data. The Cole-Kripke algorithm computes a weighted sum of activity (movement counts) within the current minute, the preceding 4 minutes, and the following 2 minutes to score each epoch as awake (up period) or asleep (down period).^29^ The variables used in the present study were: mean activity during sleep (derived from the total number of movement counts during the down period divided by the number of 60-second epochs), minutes spent awake during the down period, sleep latency (the time taken to fall asleep), sleep efficiency (percentage of down period spent asleep after removing sleep latency), wake after sleep onset (minutes spent awake during the down period after removing sleep latency), and sleep fragmentation (number of awakenings/total minutes of sleep × 100). These are the variables typically reported when using actigraphs.^26–28^

#### Statistical Analyses

The externalizing behavior scale was positively skewed (skew = 1.51). Log transformation improved the skew of the variable (log transform skew = −0.35). No major differences were found when rerunning analyses using the transformed version of the variable, so the nontransformed results are reported here. A multiple regression was used to test the hypotheses that externalizing behaviors would be associated with poor self-reported sleep quality and that callous-unemotional traits would not. Six independent multiple regression models were performed to test the same hypothesis (that externalizing behaviors would be associated with poor sleep quality, whereas callous-unemotional traits would show no relationship) for each of the objective measures of sleep derived from the actigraphy data. For the analysis of objective sleep quality, the normal significance level of *p* < .05 was reported. However, as multiple models are presented, the conservative Bonferroni corrected significance level is also provided, set at *p* < .008 (calculated as .05/6).

## RESULTS

### Descriptive Statistics and Correlations

Descriptive statistics and correlations between all variables are shown in Table 3. There were significant positive associations between externalizing behaviors and callous-unemotional traits (*r* = 0.37, *p* < .001). Externalizing behaviors were significantly positively associated with poor self-reported sleep quality (*r* = 0.25, *p* < .001). Callous-unemotional traits were not correlated with self-reported sleep quality (*r* = −0.04, *p* = .43).

**Table 3:** Descriptive Statistics and Correlations for Key Variables in Study 2.

	Variable	Mean	SD	Correlations							
				1	2	3	4	5	6	7	8
1	Externalizing behaviors	7.02	5.98								
2	Callous-unemotional traits	30.28	7.61	0.37***							
3	Self-reported sleep quality	6.85	3.52	0.25***	−0.04						
4	Mean activity	15.77	8.02	−0.25	−0.41**	0.34*					
5	Wake minutes	63.04	33.68	−0.18	−0.31*	0.35*	0.90***				
6	Sleep efficiency	89.00	6.61	0.14	0.39*	−0.24	−0.85***	−0.88***			
7	Sleep latency	13.19	16.30	−0.21	−0.16	0.36*	0.46**	0.43**	−0.10		
8	Wake after sleep onset	52.17	29.45	−0.08	−0.26	0.21	0.77***	0.90***	−0.94***	0.02	
9	Sleep fragmentation	3.73	1.92	0.04	−0.34*	0.21	0.60***	0.75***	−0.83***	0.11	0.81***

SD = standard deviation.

Externalizing behaviors (adult self-report) possible range = 0–50. Higher score = higher externalizing behaviors.

Callous-unemotional traits (Levenson’s self-report primary callous-unemotional traits subscale) possible range = 0–64. Higher score = higher callous-unemotional traits.

Self-reported sleep quality (Pittsburgh Sleep Quality Index) possible range = 0–21. Higher score = poorer sleep.

Self-reported sleep quality data collected from *N* = 338 participants.

Objective sleep quality (data collected from a subset [n = 43] of participants): On variables 4, 5, 7, 8, and 9 a higher score = worse sleep quality, and on variable 6 a higher score = better sleep quality.

*p < .05, **p < .01, ***p < .001.

Externalizing behaviors were not significantly associated with any of the objective measures of sleep. Callous-unemotional traits were significantly positively correlated with one actigraphy measure (sleep efficiency) and significantly negatively correlated with three actigraphy measures (mean activity, wake minutes, and sleep fragmentation), all indicative of better sleep quality (see Table 3).

### Regression Analyses

After controlling for age and sex, higher externalizing behaviors were related to poorer self-reported sleep quality, and higher callous-unemotional traits were associated with better self-reported sleep quality independently from age and sex. The regression results are shown in Table 4. The same analysis was also performed on just the subsample who also wore actigraphs (*n* = 43), and a similar pattern of results emerged. Although the magnitude and direction of effects were similar for both externalizing behaviors (*β* = 0.20, *p* = .28) and callous-unemotional traits (*β* = −0.21, *p* = .22) with regard to sleep quality, the results were not statistically significant. This is likely due to low statistical power.

**Table 4:** A Series of Multiple Regressions Exploring the Relationship Between Externalizing Behaviors, Callous-Unemotional Traits, and Self-Reported and Objective Measures of Sleep Quality in Study 2.

Predictor variables	Dependent variables													
	Self-reported sleep quality		Mean activity		Wake minutes		Sleep efficiency		Sleep latency		WASO		Sleep fragmentation	
	*β*	95% CI	*β*	95% CI	*β*	95% CI	*β*	95% CI	*β*	95% CI	*β*	95% CI	*β*	95% CI
Age	0.01	−0.01 to 0.02	−0.12*	−0.21 to −02	−0.12*	−0.22 to −0.02	0.09	−0.01 to 0.18	−0.11*	−0.21 to −0.01	−0.08	−0.19 to 0.02	−0.05	−0.15 to 0.05
Sex	0.19	−0.04 to 0.42	−0.38	−1.06 to 0.30	−0.57	−1.28 to 0.14	0.70	−0.01 to 1.41	−0.50	−1.25 to 0.25	−0.46	−1.22 to 0.30	−0.71	−1.44 to 0.02
Externalizing behaviors	0.32***	0.20 to 0.43	−0.17	−0.55 to 0.21	−0.12	−0.52 to 0.28	0.06	−0.33 to 0.46	−0.21	−0.63 to 0.21	−0.01	−0.44 to 0.42	0.14	−0.26 to 0.55
Callous- unemotional Traits	−0.15*	−0.26 to −0.03	−0.57**	−0.92 to −0.21	−0.47*	−0.84 to −0.10	0.55**	0.19 to 0.92	−0.26	−0.65 to 0.13	−0.39	−0.78 to 0.01	−0.51*	−0.88 to −0.13
*F*(4, 323) (4, 38)	8.16***		4.30**		3.09*		3.31*		1.85		1.51		2.53	
Adjusted *R*^2^	0.08		0.24		0.17		0.18		0.08		0.05		0.13	

Self-reported measures of sleep collected from *N* = 338 participants. Objective measures of sleep collected from a subset (*n* = 43) of participants.

CI = confidence intervals, WASO = wake after sleep onset.

Sex coded as 1 = male, 2 = female.

*p < .05, **p < .01, ***p < .001.

There were no significant relationships between externalizing behaviors and any of the objective measures of sleep. Callous-unemotional traits predicted better sleep quality assessed using certain objective measures (Table 4). Specifically, higher levels of callous-unemotional traits were associated with less mean activity during down time, fewer minutes spent awake during down time, greater sleep efficiency, and less fragmented sleep. Using the Bonferonni corrected significance threshold of *p* < .008, lower mean activity and greater sleep efficiency remained significantly associated with higher levels of callous-unemotional traits. The interaction between externalizing behaviors and callous-unemotional traits was not examined in study 2 due to the smaller sample size.^30^

## DISCUSSION

This is the first study to our knowledge to investigate the association between sleep quality and both externalizing behaviors and callous-unemotional traits. Our results were largely in line with our hypotheses. First, in support of previous studies,^1–3^ we found a small but significant association between externalizing behaviors and poorer sleep quality using self-report in two separate studies. The association between externalizing behaviors and poorer self-reported sleep quality was partially mediated by anxiety in the first study. We were unable to run this analysis in the second study due to the smaller sample size in study 2^24^. Unexpectedly, we did not find an association between externalizing behaviors and sleep quality when the latter was measured objectively using actigraphy. Second, in line with expectations, we did not find a significant association between callous-unemotional traits and self-reportedly rated sleep quality in two separate studies, and in fact a relationship between callous-unemotional traits and better self-reported sleep quality was found in one of the samples. Strikingly, when sleep quality was assessed objectively using actigraphy, callous unemotional traits were associated with better sleep quality. Finally, we showed that levels of callous-unemotional traits moderated the relationship between externalizing behaviors and self-reported sleep quality, with high levels of callous-unemotional traits associated with a weaker relationship between externalizing behaviors and sleep.

### Externalizing Behaviors and Sleep Quality

The finding that there was an association between higher levels of externalizing behaviors and poorer sleep quality is in agreement with previous work reporting this link.^1–3^ The modest magnitude of the association also fits well with the prevailing evidence that this association is weaker in magnitude than that between sleep and other variables such as depression,^7^ with which disturbed sleep is so commonly linked that it is listed within the diagnostic criteria.^31^

The absence of an association between externalizing behaviors and objective sleep quality assessed using actigraphy needs to be addressed. Although there was some correspondence between self-reported and objective measures of sleep in the current study, associations were moderate, suggesting that these measures also tap into different components of sleep.^32^ This is unsurprising as while the PSQI contains items on sleep disturbance, it also assesses other aspects of sleep that are not possible to assess using actigraphy such as daytime dysfunction and use of sleep medication. Others have suggested that whereas actigraphy can provide a reliable measure of sleep schedule and sleep periods, the correspondence between actigraphic sleep quality measures (eg, wake after sleep onset, sleep efficiency) and self-reportedly related sleep quality is relatively poor.^26^ It is not uncommon to find a misalignment of results when using self-reported and objective measures of sleep—with clear examples coming from the sleep/depression literature in youth^13^ and in the case of those with paradoxical insomnia.^33^

One potential explanation for the discrepancy between results obtained using self-reported and objective measures could be due to the differing samples used. Actigraphic data were only collected from a smaller subsample of the participants in Study 2 that may have differed in important ways (eg, participants who took part in actigraphy had a smaller age range) from the full sample in Study 2. However, the same patterns of results regarding self-reported measures of sleep quality were found when the analyses were run only on the subsample who also completed actigraphy, that is, externalizing behaviors were associated with poorer self-reported sleep quality, and callous-unemotional traits were associated with better self-reported sleep quality (though the results did not reach significance, likely due to low statistical power).

### Anxiety As a Mediator of the Associations Between Externalizing Behaviors and Sleep Quality

In order to move forward in our understanding of the association between externalizing behaviors and sleep quality, the mechanisms linking these variables need to be established. Here we examined one candidate, finding that anxiety partially mediated the association. This is the first study to examine such a link. One potential mechanism for this mediation is that individuals who engage in externalizing behaviors later internalize the experience which leads to anxious thoughts about the behaviors committed.^34^ This in turn could lead to problems with sleep.^3,7,35^ Other possible mediators and moderators of the association between these phenotypes should be examined in future studies.

### Callous-Unemotional Traits and Sleep Quality

Among our most novel findings is the absence of an association between callous-unemotional traits and poor sleep quality. This is particularly striking as disturbed sleep or sleep of poor quality seems to be associated with many different aspects of our functioning—including a range of psychiatric variables (such as depression, anxiety, schizophrenia, obsessive compulsive disorder, and post-traumatic stress disorder) examined previously.^7,13^ The finding that callous-unemotional traits are associated with better sleep quality when assessed objectively is even more noteworthy—although this makes sense when considering the nature of the callous-unemotional phenotype, which involves a lack of guilt and remorse for example,^11^ which may also mean lower levels of rumination, catastrophizing, and other processes that could disturb sleep.^5^ This association also fits well in terms of other characteristics often associated with callous-unemotional traits, including low levels of anxiety and attenuated reactivity of the brain’s affective circuit to emotional stimuli^12^—again, which may also be conducive to good sleep quality. As such, callous-unemotional traits without anxiety could be described as providing a protective factor for the negative impact of externalizing behaviors on sleep.

It is worth considering the high correlations between various actigraphy measures used in this study (see Table 3). This raises the question of whether these variables should be thought of as separate measures. Results showed lower mean activity and greater sleep efficiency to be associated with callous-unemotional traits. However, the correlation between these two measures was very high (r = −0.85). As such, rather than considering these as distinctly separate measures, it may be more sensible to interpret this finding more generally as a significant association between lower activity during the night/more sleep and callous-unemotional traits rather than try to consider these measures as separate constructs. Sleep latency, a measure of sleep initiation, was in contrast, not significantly associated with callous-unemotional traits.

### Limitations

The studies presented have a number of strengths, including the novelty of the questions addressed, the use of two data sets to address our questions, and the use of both self-reported and objective measures of sleep. Nonetheless, these results need to be considered alongside limitations. First, actigraphy was only collected from a (*n* = 43) subsample of participants who took part in Study 2, and it is possible that some differences may exist between this subsample and the whole sample (eg, participants who took part in actigraphy had a smaller age range). This needs to be considered when interpreting the results of this work. Second, cross-sectional data were used in these analyses—and hence, it was not possible to establish the direction of effects between the variables. Although sleep quality was the dependent variable in this study, it is equally likely that poor sleep could lead to externalizing behaviors.^36^ Indeed, poor sleep has been associated with compromised emotional regulation^37^ which could explain some of the associations found in this study. Further prospective longitudinal data is needed to establish the direction of effects.

Third, we need to consider the representativeness of the data sets. For example, the G1219 sample comprises twins and nontwin sibling pairs. However, we are not aware of evidence to suggest that antisocial behavior or sleep is markedly different in adult twins as compared to adult nontwins. The G1219 participants were also from more educated families than the general population.^21^ Furthermore, this study has been running for many years, and attrition has inevitably occurred over waves of data collection. Attrition analyses demonstrated that participants staying in this study up until wave 4 (reported here) were more likely than those leaving the study to be female.^21^ Although mean levels may be different in these samples compared to the general population, there is no evidence to suggest that the relationships between these variables would differ in our samples compared to the general population. Also, the G1219 sample consists mostly of young adults (age range 18–27 years), therefore, it is unclear whether the results obtained here can be generalized to other age groups.

The measures in Study 1 were administered as part of a longitudinal data collection that focused on multiple measures.^38^ As such, this restricted the length of what could be included in order to help reduce costs on participants taking part in a large scale longitudinal study. Therefore, some items from existing measures had to be removed, as is often the case in studies of this type. As a result, the reliability of these measures may have been affected and this should be considered when interpreting the results. Although it should be noted that the Cronbach’s alphas for these adapted measures were still considered to be modest/good. It is also worth noting that the measures used to assess callous-unemotional traits in the two samples differed. This could explain why Study 1 found no relationship between sleep quality and callous-unemotional traits and Study 2 found callous-unemotional traits to be related to better sleep quality.

Study 2 collected self-reported data via the internet. Although this is now considered to be a reasonable approach to data collection,^39^ a disadvantage is that it is difficult to ensure that participants were optimally responding to the items (eg, if they were giving questions their full attention, fully understanding what the questions were asking). Relatedly, in both studies, self-report was used to measure phenotypes. A more thorough investigation could have assessed traits from other raters (eg, family members, spouses) along with the participants own self-report.

### Practical Implications

It is premature to suggest practical implications of this work as we did not focus on a sample with extreme scores on the variables being assessed, but it is possible that by addressing anxiety, we could weaken the link between externalizing behaviors and sleep quality; or that by improving sleep we could reduce externalizing behaviors. The latter suggestion fits well with other research showing that improving sleep can have positive implications for a whole host of difficulties.^13^ In fact, there is preliminary data, albeit focusing on different sleep problems and age groups, to show, more directly, that treating sleep can have a positive impact on aggression.^40,41^

Although externalizing behaviors are linked to poorer sleep quality in this study, the same does not hold for callous-unemotional traits, where an association with better sleep is seen. This supports the idea that different forms of antisocial behavior (externalizing behaviors and callous-unemotional traits) should perhaps be treated in different ways.^42,43^ Overall, this work shows that poor sleep is not consistently associated with all forms of psychopathology and “negative outcomes”. Instead, we need to keep in mind that the nature of and mechanisms underlying specific associations, and the potential impact of protective factors, are yet to be thoroughly determined.^13^

## FUNDING

TAM is supported by a Sir Henry Dale Fellowship jointly funded by the Wellcome Trust and the Royal Society (Grant Number 107706/Z/15/Z). DD was supported by an Economic and Social Research Council Advanced Quantitative Methods PhD studentship (ES/J500215/1). EV is a Royal Society Wolfson Research Merit Award Holder.

## FINANCIAL SUPPORT

**G1219 study:** Waves 1–3 funded by the W T Grant Foundation, the University of London Central Research fund and a Medical Research Council Training Fellowship (G81/343) and Career Development Award (G120/635) to Thalia C. Eley. Wave 4 supported by the Economic and Social Research Council (RES-000-22-2206) and the Institute of Social Psychiatry (06/07 – 11) to Alice M. Gregory. Wave 5 supported by funding from Goldsmiths, University of London to Alice M. Gregory.

**Second study:** Actiwatches were purchased with a Goldsmiths Pump Priming Grant to Alice M. Gregory.

## AUTHORSHIP RESPONSIBILITIES

DD conducted the statistical analyses and led the write-up of the study. RA, BH, CM, JP, NB, ROL helped with data collection for the second study. GC and AS provided expertise in considering the phenotypes in this study. TCE is the founder of the G1219 study. BM, RR and TAM are part of the G1219 team with expertise in externalizing disorders. EV and AMG supervised this paper and wrote sections of this manuscript. All authors contributed to the drafting of this manuscript.

## INSTITUTION AT WHICH WORK WAS PERFORMED

Goldsmiths, University of London and the Institute of Psychiatry, Psychology and Neuroscience, King’s College London.

## DISCLOSURE STATEMENT

AMG has provided guidance and educational content for a freely available educational website focused on infant sleep. This website is partially supported by Johnson and Johnson, but they do not have any influence over content and do not advertise on it. She also contributes to BBC Focus Magazine.
